# Transcriptome analysis of Sacha Inchi (*Plukenetia volubilis* L*.*) seeds at two developmental stages

**DOI:** 10.1186/1471-2164-13-716

**Published:** 2012-12-20

**Authors:** Xiaojuan Wang, Ronghua Xu, Ruling Wang, Aizhong Liu

**Affiliations:** 1 Key Laboratory of Tropical Plant Resource Science, Xishuangbanna Tropical Botanical Garden, Chinese Academy of Sciences, 88 Xuefu Road, Kunming, 650223, China; 2 Kunming Institute of Botany, Chinese Academy of Sciences, 132 Lanhei Road, Kunming, 650201, China; 3 Graduate University of Chinese Academy of Sciences, Beijing, 100049, China

**Keywords:** Transcriptome, Unsaturated fatty acids, Omega-3 fatty acids, Triacylglycerols, Gene expression

## Abstract

**Background:**

Sacha Inchi (*Plukenetia volubilis* L., Euphorbiaceae) is a potential oilseed crop because the seeds of this plant are rich in unsaturated fatty acids (FAs). In particular, the fatty acid composition of its seed oil differs markedly in containing large quantities of α-linolenic acid (18C:3, a kind of ω-3 FAs). However, little is known about the molecular mechanisms responsible for biosynthesis of unsaturated fatty acids in the developing seeds of this species. Transcriptome data are needed to better understand these mechanisms.

**Results:**

In this study, *de novo* transcriptome assembly and gene expression analysis were performed using Illumina sequencing technology. A total of 52.6 million 90-bp paired-end reads were generated from two libraries constructed at the initial stage and fast oil accumulation stage of seed development. These reads were assembled into 70,392 unigenes; 22,179 unigenes showed a 2-fold or greater expression difference between the two libraries. Using this data we identified unigenes that may be involved in *de novo* FA and triacylglycerol biosynthesis. In particular, a number of unigenes encoding desaturase for formation of unsaturated fatty acids with high expression levels in the fast oil accumulation stage compared with the initial stage of seed development were identified.

**Conclusions:**

This study provides the first comprehensive dataset characterizing Sacha Inchi gene expression at the transcriptional level. These data provide the foundation for further studies on molecular mechanisms underlying oil accumulation and PUFA biosynthesis in Sacha Inchi seeds. Our analyses facilitate understanding of the molecular mechanisms responsible for the high unsaturated fatty acids (especially α-linolenic acid) accumulation in Sacha Inchi seeds.

## Background

Sacha Inchi (*Plukenetia volubilis* L.), also known as Inca Inchi or mountain peanut, belongs to the family Euphorbiaceae. It is a perennial oleaginous woody vine indigenous to tropical Peruvian jungles that lie at altitudes between 200 and 1500 m. This legume has a star-shaped fruit, which contains dark oval seeds characterized by high levels of protein (ca. 30%) and oil (ca. 50%) [1,2]. Its flour and oil are consumed by Peruvian Indians, who have used them to prepare foods and beverages for hundreds of years [3]. The fatty acid (FA) composition of Sacha Inchi seed oil is distinctive because its seeds contain a large amount of polyunsaturated fatty acids (PUFAs) comprising about 93% of total FAs. Specifically, α-linolenic acid (18C:3 cis Δ9, 12, 15, a kind of ω-3 FAs) comprises ca. 50% of the FAs content, and linoleic acid (18C:2 cis Δ9, 12, a kind of ω-6 FAs) comprises ca. 35%. Other FAs such as palmitic acid (16C:0), stearic acid (18C:0), and oleic acid (18C:1cisΔ9) are present in small proportions [1,2]. 

Because PUFAs such as α-linolenic and linoleic acids cannot be synthesized in mammals, they are essential FAs (i.e., required in the diet) [4,5]. These PUFAs not only exert a hypocholesterolemic effect against coronary heart disease and hypertension when used as food supplements, but also are critical for the development of infants during pregnancy and breastfeeding periods [6-8]. The recommended ω-6: ω-3 FA ratio in the human diet is approximately 2:1 to 6:1 [9,10]; however, traditional oil seed crops (e.g., soybean, peanut, maize, sunflower, and rape) have relatively low α-linolenic content (below 10%). The high ω-6: ω-3 FA ratio in our typical diet (approximately 15:1) is thought to be a major contributor to cardiovascular disease [9]. In contrast, Sacha Inchi seed oil has an approximately 7:10 ratio of ω-6: ω-3 FAs. The physiological mechanisms responsible for ω-3 FAs biosynthesis in the seeds of Sacha Inchi are unknown. Understanding the molecular mechanisms underlying seed development and lipid biosynthesis in Sacha Inchi may provide new insights for isolating novel genes and serve the use of genetic engineering to increase the α-linolenic content of traditional vegetable oils.

Generally, two main pathways are involved in storage lipid accumulation: FA biosynthesis and triacylglycerol (TAG) assembly. In all plants studied, acyl carrier protein (ACP)-dependent *de novo* FA synthesis occurs almost exclusively in plastids [11,12]. FAs are synthesized from acetyl-CoA in a three-step process: (a) irreversible carboxylation of acetyl-CoA by acetyl-CoA carboxylase to form malonyl-CoA; (b) repeated condensation of malonyl-CoA with a growing ACP-bound acyl chain by FA synthase complex, consecutively adding two carbon units to form 16:0-ACP; and (c) elongation and desaturation of 16:0-ACP to form 18:0-ACP and 18:1-ACP, respectively [13]. Desaturation of 18:0-ACP to 18:1-ACP catalyzed by a stromal stearoyl-ACP desaturase is one of the key steps regulating unsaturated FA levels in the cell. In oilseeds, more than 95% of newly synthesized FAs [18C:1 > > 16C:0 > 18C:0] are exported from plastids as CoA thioesters to enter the eukaryotic glycerolipid synthesis pathway [14,15]. In the endoplasmic reticulum, acyl-CoAs can be used for the sequential sn-1 and sn-2 acylation of glycerol-3-phosphate to produce phosphatidic acid, and are then converted to 1, 2-sn-diacylglycerol (DAG) by phosphatidate phosphatase. The third acyl-CoA-dependent acylation catalyzed by DAG acyltransferase leads to the production of TAG (i.e., FA incorporation onto the glycerol backbone known as the Kennedy pathway) [16]. TAG can also be formed by acyl-CoA-independent reactions using DAG and phosphatidylcholine (PC) as acyl donors [17,18]. Phospholipid:DGAT (diacylglycerol acyltransferase) catalyzes the acyl transfer from PC to DAG to form lysophosphatidylcholine and TAG, whereas diacylglycerol transacylase catalyzes the transfer of an acyl moiety between two DAG molecules to form TAG and monoacylglycerol [13]. In the mature oilseed, TAG is stored in densely packed oil bodies surrounded by oleosin protein [19,20]. Usually, oil accumulation in developing seeds is associated with an increase in the number of oil bodies.

In oil-accumulating cells of plant seeds, 18C:1 is desaturated to 18C:2 and 18C:3 by two microsomal desaturase enzymes, FAD2 and FAD3 [21,22]. Extraplastidic desaturation of 18C:1 to 18C:2 and 18C:3 arises while the FAs are bound to PC [23], the major site of FA desaturation in eukaryotes [24]. In current models of seed lipid biosynthesis, 18C:1 is incorporated into PC by one of two routes. Direct incorporation from 18C:1-CoA exported by the plastids likely occurs through the action of acyl-CoA: lysophosphatidylcholine acyltransferase [25,26]. Alternatively, 18C:1 may be incorporated into DAG by the Kennedy pathway, after which 18C:1-DAG is converted to PC by cytidine 5'-diphosphocholine: diacylglycerol cholinephosphotransferase [27-29]. Oil-accumulating tissues use different strategies to enrich PUFAs in TAG. For example, converting PC to DAG (for TAG synthesis) may be accomplished by the reverse action of cholinephosphotransferase or by phosphatidylcholine: diacylglycerol cholinephosphotransferase [29-31]. Alternatively, PUFAs may be enriched in TAG by PC acyl editing that incorporates nascent 18C:1 into PC and releases PUFAs, generating an acyl-CoA pool of newly synthesized FAs and further-modified FAs for glycerolipid synthesis in the endoplasmic reticulum [32]. Mechanisms for PUFA biosynthesis and accumulation are not known for most oilseeds, nor are the enzymes/genes that differ between the pathways [33]. Because Sacha Inchi seed oil contains uncommonly high levels of ω-3 FAs, it is a good model to dissect metabolic pathways involved in oil and PUFA biosynthesis.

The development of high-throughput sequencing technologies has enabled the efficient and economical resequencing of entire genomes or sampling of entire transcriptomes. For example, Illumina sequencing technology produces millions of short cDNA reads from a single instrument run, which consists of the transcription level for each gene. It determines absolute rather than relative gene expression, thus providing greater insight and accuracy than microarray analysis [34-36]. *De novo* transcriptome sequencing and characterization based on Illumina second-generation sequencing technology has enabled the rapid identification and profiling of differentially expressed genes in the sweet potato [37], eucalyptus tree [38], chickpea [39], orchid [40], taxus [41], sesame [42], and peanut [43]. However, transcriptomic information is lacking for Sacha Inchi. We were interested in identifying genes involved in PUFA biosynthesis (especially α-linolenic acid) in Sacha Inchi seeds during the period of fast oil accumulation; therefore, transcriptome analysis was carried out during two stages of seed development by using an Illumina paired-end sequencing strategy. In this study, we generated more than four billion bases of high-quality DNA sequence and obtained 70,392 unigenes from the Sacha Inchi seed transcriptome. Among them, 22,179 unigenes showed a 2-fold or greater expression difference between the two seed development stages. The assembled, annotated transcriptome sequences and gene expression profiles provide useful information for the identification of genes involved in unsaturated FAs and TAG biosynthesis.

## Results and Discussion

### Illumina paired-end sequencing and *de novo* assembly

**Figure 1:**
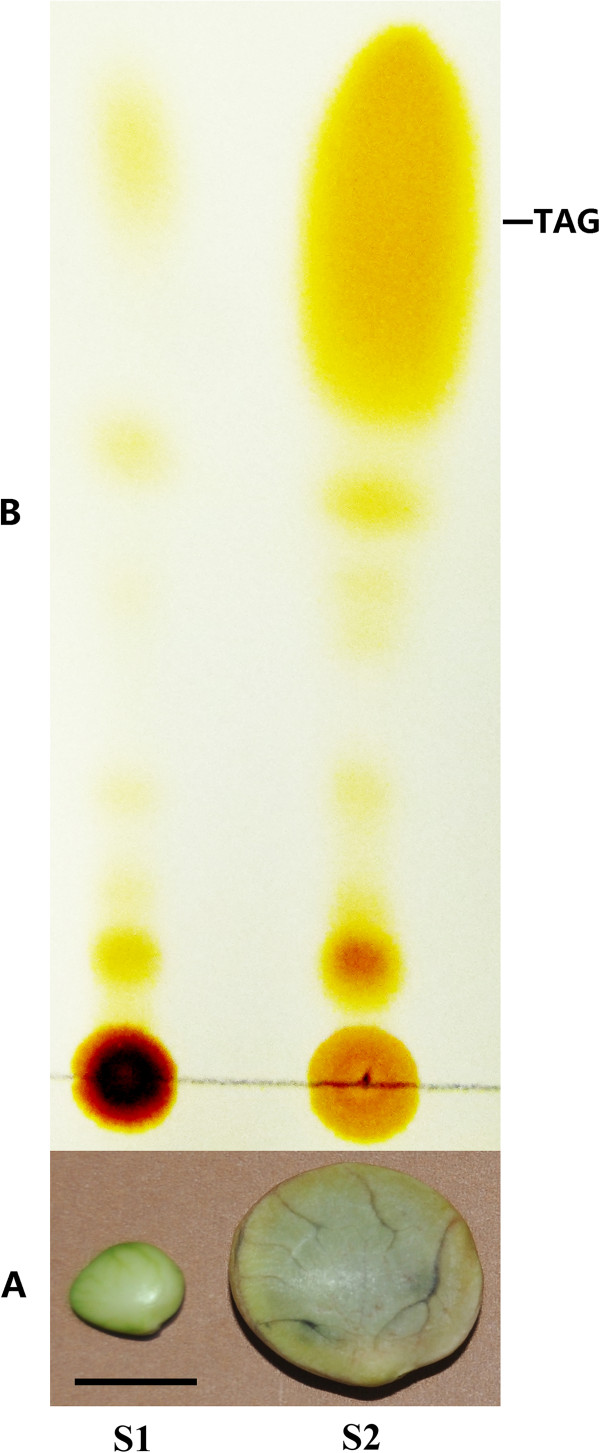
**Sacha inchi seeds at two different developmental stages and analysis of triacylglycerols (TAGs).** (**A**) **S1**: Sacha Inchi seed harvested at 10 days after pollination (DAP), **S2**: Sacha Inchi seed harvested at 60 DAP, scale bar = 1.0 cm. (**B**) Thin layer chromatography analysis of TAGs isolated from seeds at the initial stage (**S1**) and fast oil accumulation stage (**S2**).

The two cDNA libraries were constructed from two stages of developing seeds (i.e., the initial stage, S1 and the fast oil accumulation stage, S2) (Figure 1) and sequenced by Illumina high-throughput sequencing platform. We obtained a total of 52.6 million 90-bp paired-end reads from two libraries (26.3 million for each), encompassing 2.36 Gb of the sequence data for each library (Table 1). After stringent quality assessment and data filtering, 25.1 and 25.4 million reads with a base quality score greater than 20 were selected for further analysis and deposited in the National Center for Biotechnology Information (NCBI) Short Read Archive (accession number: SRA062070). The GC contents of the two libraries were 43.99% (S1) and 44.61% (S2) (Table 1), respectively.

**Table 1:** Summary statistics of clean reads in two cDNA libraries from Sacha Inchi seeds

	**S1 Initial stage (5–10 DAP) library**	**S2 Fast stage (50–65 DAP) library**
Total reads	26,266,670	26,266,670
Total Nucleotides (nt)	2,364,000,300	2,364,000,300
High-quality reads	25,097,804	25,370,976
High-quality reads (%)	95.55%	96.59%
Low quality reads	1,168,866	895,694
Low quality reads (%)	4.45%	3.41%
GC percentage of high-quality reads (%)	43.99%	44.61%

**Figure 2:**
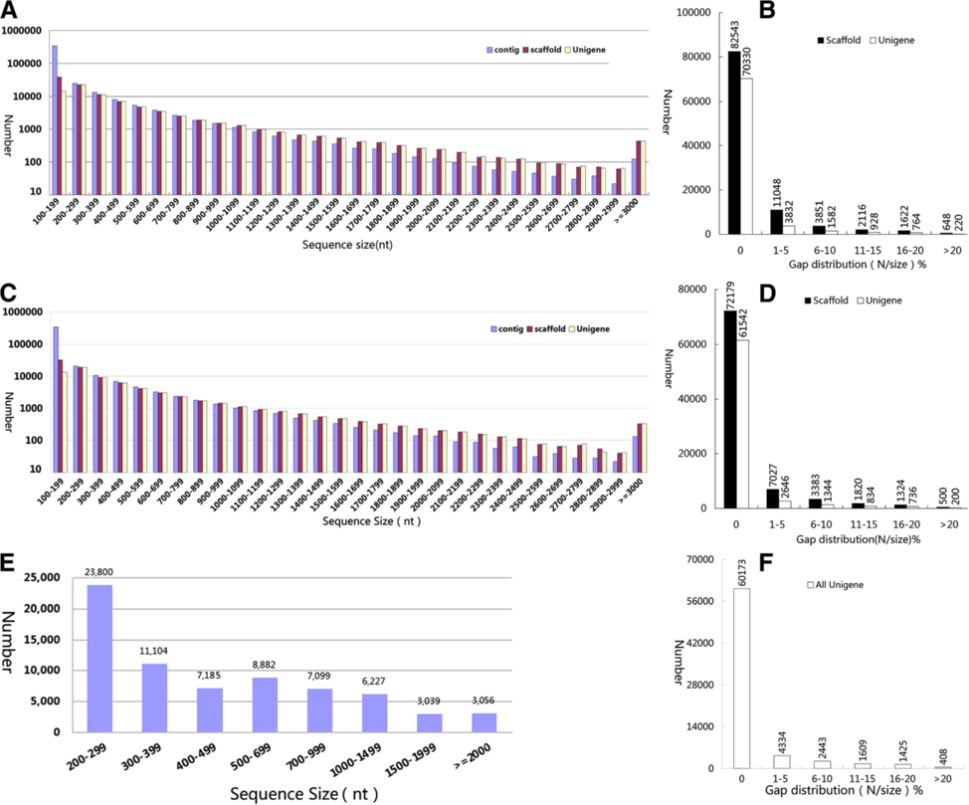
**Analysis of Illumina short read assembly quality.** Size distributions of *de novo* assembled contigs, scaffolds, and unigenes are shown for the (**A**) initial stage (S1) and (**C**) fast oil accumulation stage (S2) of seed development. Gap distributions of assembled scaffolds and unigenes are shown for the (**B**) initial stage (S1) and (**D**) fast oil accumulation stage (S2) of seed development. Size and gap distributions of the nonredundant unigenes are shown for the (**E**) initial stage (S1) and (**F**) fast oil accumulation stage (S2) of seed development. Gap distribution (N/size) %: gap percentage (N amount/sequence length) distribution.

The SOAPdenovo assembly program developed specifically for use with high-throughput short-read sequences [44] was used for *de novo* assembly. From the high-quality reads in the S1 library, 352,172 contigs with a median length of 234 bp were assembled (Figure 2A). After paired-end joining and gap filling by SOAPdenovo, the contigs were assembled into 101,828 scaffolds with a median length of 597 bp (Figure 2A). Although 81.06% scaffolds had no gaps, roughly 0.51 Mb gaps (1.24% of total scaffold sequences) remained unclosed (Figure 2B). To further shorten the remaining gaps, paired-end reads were gathered with one end mapped on the unique contig and the other located in the gap region to fill small gaps within the scaffolds. Sequences containing the least Ns and that could not be extended on either end were defined as unigenes. The scaffolds were further clustered into 77,656 unigenes with a median length of 660 bp (Figure 2A). In this step, nearly half of the gaps were filled, and only 0.26 Mb gaps (0.68% of total unigene sequences) remained unclosed (Figure 2B). For the S2 library, 347,156 contigs were assembled, with a median length of 204 bp (Figure 2C). From the contigs, 86,233 scaffolds were generated, with a median length of 618 bp (Figure 2C). Although 83.70% scaffolds had no gaps, about 0.43 Mb gaps (1.21% of total scaffold sequences) remained unclosed (Figure 2D). From the scaffolds, 67,302 unigenes were obtained, with a median length of 674 bp (Figure 2C). In this step, 46.5% gaps were filled, and only 0.23 Mb gaps (0.68% of total unigene sequences) remained unclosed (Figure 2D). Finally, unigenes from the two libraries were pooled and processed to remove sequence splicing and redundancy to generate a nonredundant unigene set. In total, 70,392 nonredundant unigenes were obtained with a total length of 45.39 Mb (range 200–14,913 bp; mean 645 bp; median 929 bp). The size distribution showed that 12,322 unigenes (17.50%) were longer than 1000 bp (Figure 2E). The gap distribution of the unigenes is shown in Figure 2F. These results should provide a basis for future research such as gene cloning and transgenic studies. This Transcriptome Shotgun Assembly project has been deposited at DDBJ/EMBL/GenBank under the accession GADC00000000.

### Characterization of the nonredundant unigenes of Sacha Inchi

Of the 70,392 nonredundant unigenes, sequence directions of 44,762 unigenes were determined by performing BLASTX searches against NCBI nonredundant, Swiss-Prot, Kyoto Encyclopedia of Genes and Genomes (KEGG) database, and Clusters of Orthologous Groups protein databases with an E-value cut-off of 10^-5^ and by using ESTScan software [45]. In addition, the protein coding regions of 43,608 unigenes were predicted.

**Figure 3:**
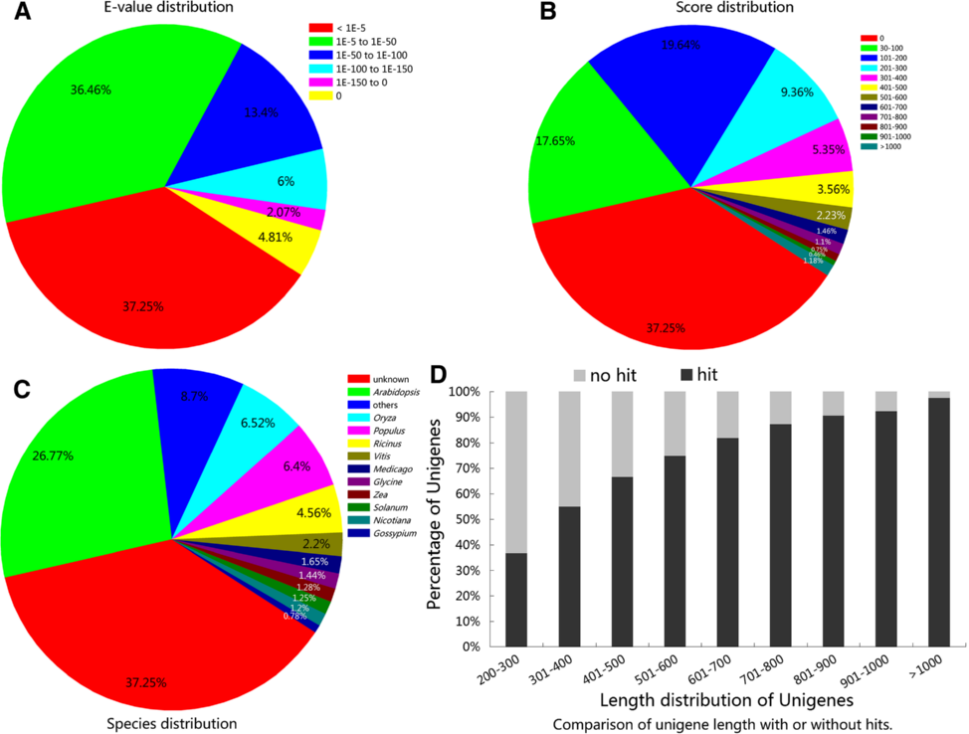
**Characteristics of homology search of assembled unigenes.** Homology search of assembled sequences was carried out by using BLASTX (cut-off E-value of 1.0E-5). When results from different protein databases conflicted, we used the following order of priority: NCBI nr, Swiss-Prot, and KEGG (**A**) E-value distribution of best BLASTX hits for each unigene. (**B**) Score distribution of the best BLASTX hit for each unigene. (**C**) Species distribution of the best BLASTX hit for each unigene. (**D**) Length of unigenes with hits compared with those without hits.

To annotate these unigenes, a homology search using the BLASTX program (E-value cut-off 10^-5^) revealed that 43,837 (62.28%), 29,714 (42.21%), and 20,142 (28.61%) of the 70,392 Sacha Inchi unigenes had significant matches with sequences in the NCBI nonredundant, SWISS-PROT, and KEGG protein databases, respectively. Altogether, 44,169 (62.75%) unigenes were successfully annotated in public databases. The E-value distribution of the top hits showed that 26.29% of the unigenes showed strong homology to previously deposited sequences (<1.0E-50), and 36.46% ranged from 1.0E-5 to 1.0E-50 (Figure 3A). Likewise, the score distribution showed that 25.46% of the unigenes had scores >200, and 37.29% had scores ranging from 30 to 200 (Figure 3B). The species distribution of the best match result for each unigene showed 26.77% matches with *Arabidopsis* sequences, 6.52% with rice (*Oryza*), 6.40% with popular (*Populus*), and 4.56% with castor bean (*Ricinus*) (Figure 3C). However, 26,223 (37.25%) unigenes (63.15% of unigenes <300 bp, and 2.54% of unigenes >1,000 bp) could not be matched to known genes (Figure 3D), suggesting that longer unigenes were more likely to have BLAST matches in the protein databases. The shorter sequences may lack a characterized protein domain or may be too short to show sequence matches, resulting in false-negative results. Because genomic and transcriptomic information is currently lacking for Sacha Inchi in databases, these unigenes without hits may be considered putative novel transcribed sequences.

### Functional classification of Sacha Inchi unigenes by Gene Ontology, Clusters of Orthologous Groups, and KEGG

**Figure 4:**
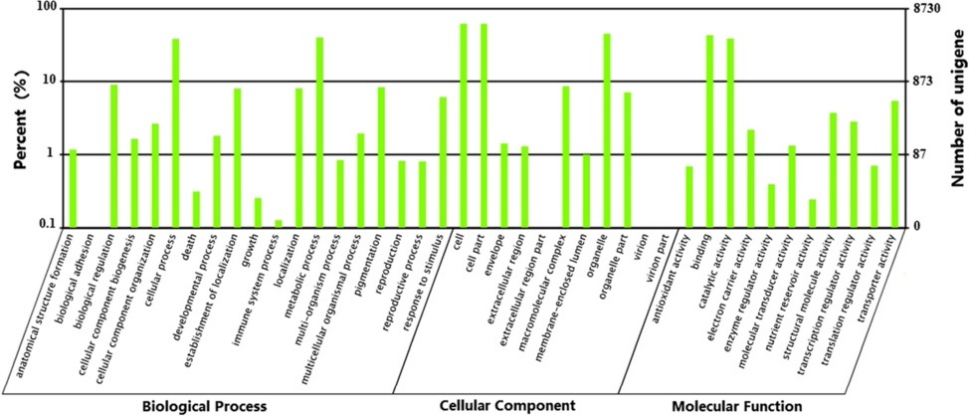
**Gene Ontology categories of assembled unigenes.** Unigenes were assigned to three categories: biological processes, cellular components, or molecular functions.

The currently available expressed sequences have been invaluable in defining the components of gene structure in Sacha Inchi. Gene Ontology assignments were used to classify functions of the predicted Sacha Inchi unigenes. Based on the Arabidopsis Information Resource Gene Ontology Slim classification provided by blast2GO [46], 8,730 unigenes were categorized into 41 functional groups under three main divisions (biological processes, cellular components, and molecular functions). In the biological processes category, metabolic processes (39.81%) and cellular processes (38.08%) were the predominant groups, followed by biological regulation (9.04%), pigmentation (8.33%), localization (8.02%), and establishment of localization (7.98%) (Figure 4). In the cellular components category, cell (61.41%) and cell part (61.40%) were the predominant groups, followed by organelles (44.71%) and macromolecular complexes (8.59%) (Figure 4). In the molecular function category, binding (42.70%) was the predominant group, followed by catalytic activity (38.60%), transporter activity (6.71%), and transcription regulator activity (5.44%) (Figure 4).

**Figure 5:**
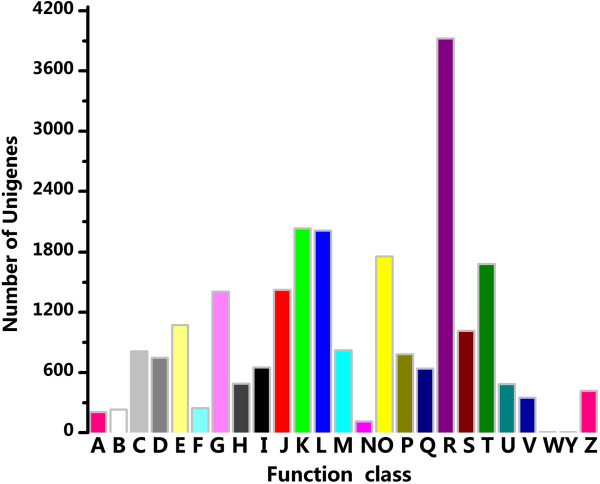
**Clusters of orthologous groups (COG) classification of assembled unigenes.** A total of 13,651 unigenes were classified into 25 functional categories according to their predicted gene products using the Cluster of Orthologous Groups (cut-off E-value of 0.00001). **A**: RNA processing and modification. **B**: Chromatin structure and dynamics. **C**: Energy production and conversion. **D**: Cell cycle control, cell division, chromosome partitioning. **E**: Amino acid transport and metabolism. **F**: Nucleotide transport and metabolism. **G**: Carbohydrate transport and metabolism. **H**: Coenzyme transport and metabolism. **I**: Lipid transport and metabolism. **J**: Translation, ribosomal structure and biogenesis. **K**: Transcription. **L**: Replication, recombination and repair. **M**: Cell wall/membrane/envelope biogenesis. **N**: Cell motility. **O**: Posttranslational modification, protein turnover, chaperones. **P**: Inorganic ion transport and metabolism. **Q**: Secondary metabolites biosynthesis, transport and catabolism. **R**: General function prediction only. **S**: Function unknown. **T**: Signal transduction mechanisms. **U**: Intracellular trafficking, secretion, and vesicular transport. **V**: Defense mechanisms. **W**: Extracellular structures. **Y**: Nuclear structure. **Z**: Cytoskeleton.

To evaluate our annotation process, the annotated unigenes were compared with the Clusters of Orthologous Groups database for functional prediction and classification. In total, 13,651 unigenes were assigned a Cluster of Orthologous Groups classification. Among the 25 categories, “transcription” represented the largest group (2,022; 14.89%), and transcripts associated with “replication, recombination and repair” (2,012; 14.74%) were most common followed by “post-translational modification, protein turnover, chaperones” (1,755; 12.86%) and “signal transduction mechanisms” (1,681; 12.31%) (Figure 5). The categories “nuclear structure” (2; 0.01%), “extracellular structures” (4; 0.03%) and “cell motility” (109; 0.80%) represented the smallest groups (Figure 5). However, categories with no concrete assignment, such as “function unknown” (7.43%) and “general function prediction only” (28.75%) accounted for a large fraction of transcripts (Figure 5).

Pathway-based analysis can further our understanding of the biological functions and interactions of genes. A total of 20,142 unigenes were assigned to 119 pathways in the KEGG database. The most represented pathways included “metabolic pathways” (4,360 unigenes), “biosynthesis of secondary metabolites pathways” (2,348 unigenes) and “plant-pathogen interaction pathways” (1,711 unigenes) (see Additional file 1). Notably, some pathways are closely linked to changes in oil content and composition that take place during Sacha Inchi seed ripening, such as “fatty acid biosynthesis pathway” (89 unigenes), “glycerolipid metabolism pathway” (146 unigenes), “linoleic acid metabolism pathway” (114 unigenes), and “α-linolenic acid metabolism pathway” (180 unigenes) (see Additional file 1). These lipid unigenes identified provided critical clues to clone and identify key functional genes involved in unsaturated FA and TAG biosynthesis in Sacha Inchi seeds.

### Analysis of differentially expressed genes at the two developmental stages

**Figure 6:**
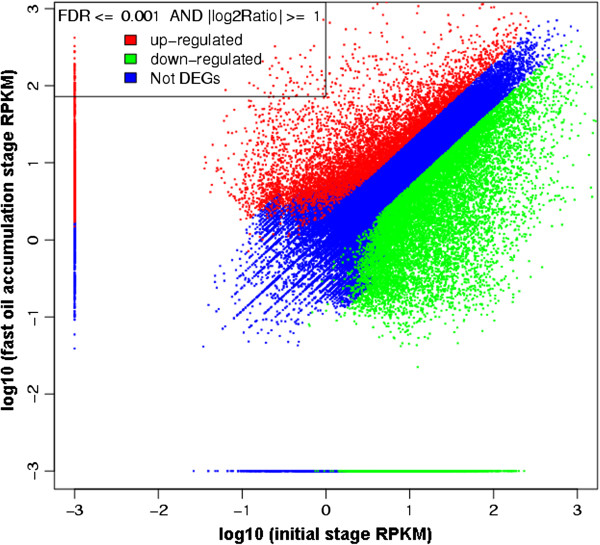
**Comparison of unigene expression between the initial stage and the fast oil accumulation stage.** Differentially expressed genes (DEGs) are shown in red and green; genes that are not differentially expressed are shown in blue. Gene expression was normalized using the RPKM method.

To identify genes showing significant changes in expression between the two libraries, the expression level of each unigene was determined by using the reads per kb per million reads (RPKM) method [36]. A general picture of the gene expression was plotted for the S1 library versus that of the S2 library (Figure 6). A total of 22,179 unigenes showed ≥2-fold or expression difference between the two libraries. Among these, 7,302 unigenes were upregulated and 14,877 unigenes were downregulated (Figure 6). The median RPKM value for all unigenes was 22.91. Of the 32 unigenes with RPKM values >1,000 in the S1 stage, we identified two different transcripts encoding a proline-rich protein that is a structural constituent of the cell wall (Table 2; unigene68591 and unigene17346) [47], one transcript encoding profilin-1 (Table 2; unigene68149), which is involved in actin cytoskeleton organization [48], and one transcript (Table 2; unigene70303) encoding arginine decarboxylase, which is thought to be required for embryogenesis [49]. These findings are consistent with rapid cell proliferation, which occurs during the early stage of seed development. Among the 32 unigenes with RPKM values >1,000 in the S2 stage, two of the most abundant transcripts code for omega-3 FA desaturase (Table 3; unigene17787 and unigene54197), reflecting the high level of α-linolenic acid in Sacha Inchi seeds. Consistent with the high levels of Asp and Glu amino acids in Sacha Inchi seeds [50], two transcripts were identified that code for glutamyl-tRNA amidotransferase subunit A and aspartyl-tRNA/glutamyl-tRNA amidotransferase subunit A (Table 3; unigene54781 and unigene54389), respectively. Transcripts encoding putative lipid transfer proteins (Table 3; unigene30721, unigene45119, unigene46454) were also abundant; these proteins are thought to participate in cutin formation, defense reactions, and response to environmental stimuli, as well as lipid transport [51]. Another abundant transcript codes for oleosin (Table 3; unigene55818), a protein thought to be involved in oil accumulation (forming “oil body”). A transcript related to seed maturation was also one the most abundant transcripts in the S2 stage (Table 3; unigene49902).

**Table 2:** Most highly expressed transcripts in the initial stage (S1) of Sacha Inchi seed development

**GeneID**	**Gene product**	**Length**	**S1 Initial stage** **(RPKM)**	**S2 Fast stage** **(RPKM)**
Unigene65859	Stellacyanin-like protein	256	7393	30
Unigene68591	Proline-rich protein	389	6375	9
Unigene70036	Beta-glucosidase	807	4501	384
Unigene60791	UV excision repair protein	629	3699	17
Unigene65952	Beta-glucosidase	259	2512	173
Unigene4436	S-adenosylmethionine synthetase	470	2295	89
Unigene24290	MLP-like protein	728	2014	7
Unigene69609	EXORDIUM LIKE 2	571	1888	24
Unigene17445	Calcium-binding protein	562	1871	11
Unigene17323	Dicyanin	714	1863	82
Unigene70303	Arginine decarboxylase	1352	1795	211
Unigene64092	Gip1-like protein	226	1652	35
Unigene66170	Gibberellin-regulated protein	265	1568	36
Unigene66806	Low temperature inducible	284	1533	24
Unigene68526	Splicing factor 1	383	1502	140
Unigene10616	Conserved hypothetical protein	773	1501	4
Unigene66001	S-adenosylmethionine synthase	260	1475	96
Unigene68224	Iron transport protein 2	359	1452	5
Unigene69618	EXORDIUM LIKE 2	573	1379	13
Unigene66078	Unknown	262	1310	112
Unigene67940	Omega-3 fatty acid desaturase, chloroplast precursor	339	1233	43
Unigene33094	Heat shock cognate 70 kDa protein	590	1222	235
Unigene68297	Iron-binding protein	364	1183	240
Unigene68149	Profilin-1	353	1154	210
Unigene17346	Proline rich protein	477	1117	97
Unigene62353	Heat-shock protein	203	1096	324
Unigene69273	Elongation factor	483	1081	33
Unigene62855	Unknown	209	1032	258
Unigene69780	Major latex-like protein	638	1026	104
Unigene69296	Gibberellin regulated protein	487	1024	22
Unigene62550	Thioredoxin h1	204	1020	181
Unigene66473	Fiber annexin	273	1005	521

**Table 3:** Most highly expressed transcripts in the fast oil accumulation stage (S2) of Sacha Inchi seed development

**GeneID**	**Gene product**	**Length**	**S1 Initial stage (RPKM)**	**S2 Fast stage (RPKM)**
Unigene49263	Ricin-agglutinin family protein	266	25	248413
Unigene40641	Preproricin	625	12	150901
Unigene40632	Preproricin	575	15	147593
Unigene51295	Unknown	307	167	5432
Unigene55818	Oleosin	715	13	4193
Unigene45984	1-cys peroxiredoxin	227	2	3015
Unigene17787	Microsomal omega-3 fatty acid desaturase	1118	11	2918
Unigene37706	BURP domain-containing protein	658	22	2549
Unigene45545	Predicted protein	222	24	2467
Unigene30721	Lipid transfer protein (LTP) family protein	325	87	2116
Unigene24053	Ricin-agglutinin family protein	492	79	2011
Unigene45119	Lipid transfer protein (LTP) family protein	217	121	1877
Unigene59025	Peroxidase	270	736	1740
Unigene54781	Glutamyl-tRNA(Gln) amidotransferase subunit A	497	108	1722
Unigene49902	Seed maturation protein PM35	276	4	1625
Unigene24688	60S ribosomal protein	442	709	1499
Unigene46988	Protein disulfide-isomerase	238	260	1482
Unigene30559	Thaumatin-like protein	425	120	1356
Unigene52866	Protein disulfide-isomerase	360	295	1330
Unigene20119	Thiazole biosynthetic enzyme	632	87	1305
Unigene52919	Protein disulfide-isomerase	362	278	1302
Unigene42819	Conserved hypothetical protein	641	3	1295
Unigene49237	Major latex allergen Hev b	266	125	1273
Unigene40227	Syringolide-induced protein	761	5	1218
Unigene54197	Endoplasmic reticulum 18:2 desaturase	442	4	1149
Unigene5950	Amidase family protein	842	75	1146
Unigene54389	Aspartyl-tRNA(Asn)/glutamyl-tRNA (Gln) amidotransferase subunit A	457	73	1139
Unigene15265	DNA binding	1294	13	1078
Unigene49312	Unknown	267	101	1046
Unigene53121	Thaumatin-like protein	372	94	1035
Unigene46454	Lipid transfer protein (LTP) family protein	232	69	1015
Unigene35810	Extensin like protein	218	4	1014

**Figure 7:**
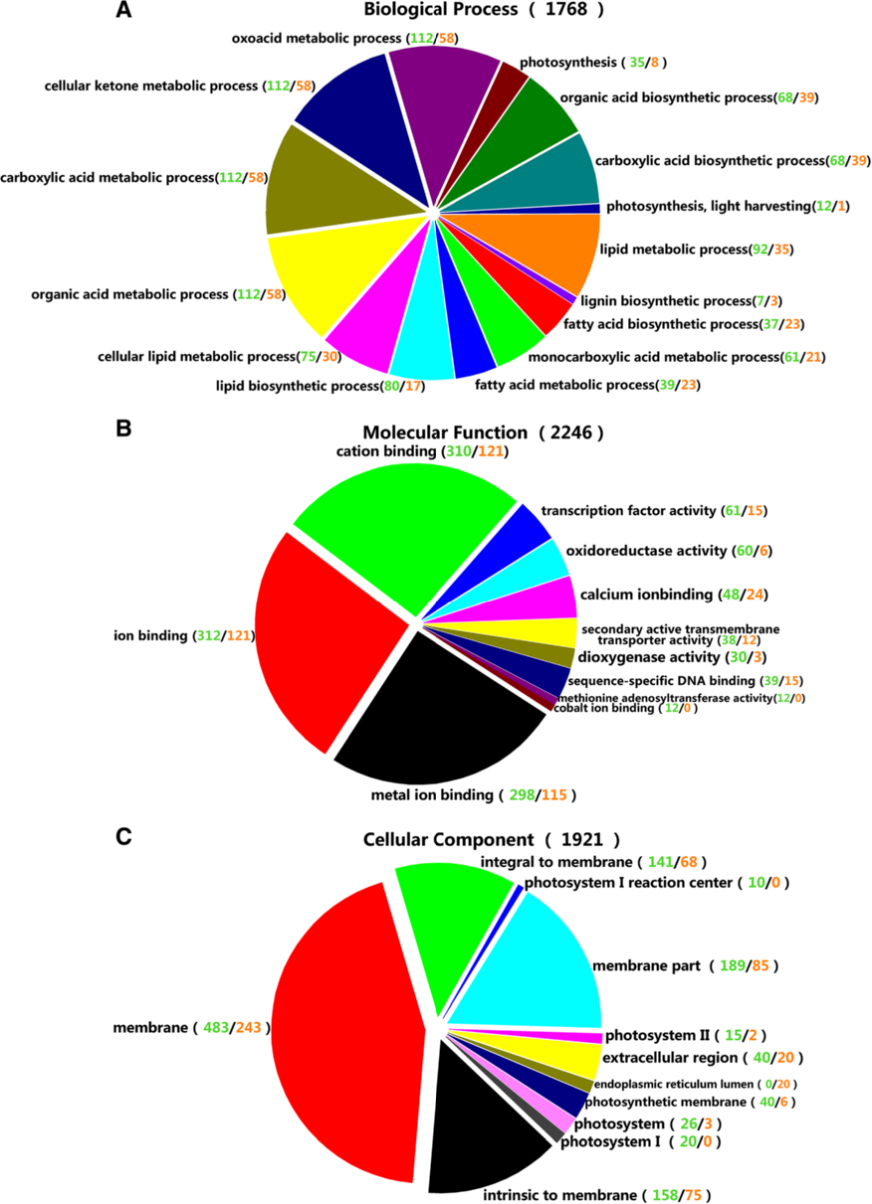
**Gene Ontology functional enrichment analysis of unigenes differentially expressed during different stages of seed development.** Unigenes were assigned to three categories: biological processes (**A**), molecular functions (**B**), and cellular components (**C**). Numbers indicate the number of unigenes that showed significant transcriptional changes in the initial stage (S1) of seed development (green) and fast oil accumulation stage (S2) of seed development (red).

To evaluate the potential functions of genes that showed significant transcriptional changes between the two libraries, Gene Ontology categories were assigned to the 3,202 differentially expressed genes. These genes are related to 18 biological processes (see Additional file 2), including cellular and metabolic processes, biological regulation, localization, establishment of localization, response to stimulus, and pigmentation. With regard to cellular components, the analysis revealed a high percentage of transcripts in the cell, cell parts, and organelle categories (see Additional file 2). The unigenes were finally classified into 11 categories based on molecular function, and the two most overrepresented terms were binding (nucleotide binding, protein binding, chromatin binding) and catalysis (see Additional file 2). The association between these terms and the lists of differentially expressed genes was tested by Gene Ontology functional enrichment analysis. The hypergeometric test was used to map differentially expressed genes to terms in the Gene Ontology database, looking for terms that were significantly enriched compared with the genome background. In the Gene Ontology enrichment, most of the gene sets upregulated in the initial stage were associated with photosynthesis (Figure 7). However, gene sets related to oil biosynthesis began to be upregulated in the fast oil accumulation stage. In particular, genes associated with the endoplasmic reticulum lumen appear to play important roles in oil biosynthesis (Figure 7).

To further understand the biological functions of differentially expressed genes, we mapped these genes to the KEGG database. In the KEGG Orthology enrichment, the most represented pathways included metabolic pathways (1,999 enzymes), biosynthesis of secondary metabolites (1,184), and plant-pathogen interactions (1,028) (see Additional file 3). Of these, pathways closely related to seed oil biosynthesis, including α-linolenic acid metabolism, linoleic acid metabolism, biosynthesis of unsaturated FAs, FA biosynthesis, and FA metabolism (see Additional file 3) may provide valuable information for the identification of novel genes involved in α-linolenic acid synthesis.

### Identification and characterization of lipid genes involved in fatty acids and triacylglycerols biosynthesis in Sacha Inchi seeds

Sacha Inchi seeds have potential applications in food and pharmaceutical industries through its ability to biosynthesize and accumulate considerable amounts of linolenic acid. The main objective of this research is to understand the mechanisms underlying lipid biosynthesis in Sacha Inchi seeds and to identify key genes involved in the biosynthesis of unsaturated FAs serving genetic engineering practice for improving seed oil quantity and quality in traditional oil crops. Expression level analysis of lipid genes between the initial and fast oil accumulation stages of Sacha inchi seed could reveal the potential critical genes underlying unsaturated FAs biosynthesis in Sacha Inchi seeds. Based on the annotation results, we summarized and compared expression levels of unigenes involved in FAs and TAG biosynthesis pathways in our two transcriptome libraries.

**Figure 8:**
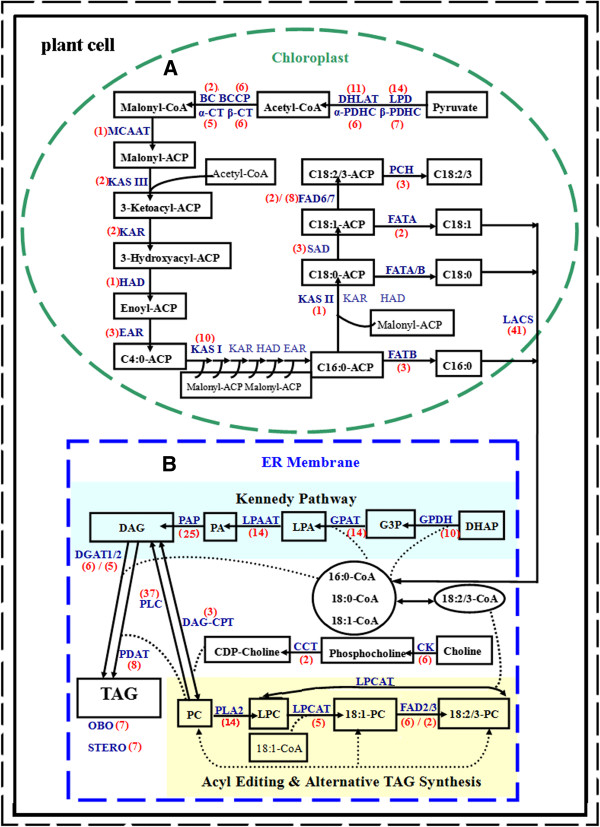
**Overview of *de novo *fatty acid and triacylglycerol(TAG) biosynthesis pathways.** Numbers shown in red font correspond to unigenes encoding enzymes in the fatty acid (**A**) and TAG (**B**) biosynthesis pathways in Sacha Inchi seeds. Lipid substrates are abbreviated: 16C:0, palmitic acid; 18C:0, stearic acid; 18C:1, oleic acid; 18C:2, linoleic acid; 18C:3, linolenic acid. Enzyme/protein abbreviations are: α-PDHC, pyruvate dehydrogenase alpha subunit; β-PDHC, pyruvate dehydrogenase beta subunit; DHLAT, dihydrolipoamide acetyltransferase; LPD, dihydrolipoamide dehydrogenase; α-CT, carboxyltransferase alpha subunit; β-CT, carboxyltransferase beta subunit; BC, biotin carboxylase; BCCP, biotin carboxyl carrier protein; MAAT, malonyl-CoA ACP transacylase; ACP, acyl carrier protein; KAS I, II, III, ketoacyl-ACP synthase I, II, III; KAS, ketoacyl-ACP synthase II; HAD, hydroxyacyl-ACP dehydrase; KAR, ketoacyl-ACP reductase; EAR, enoyl-ACP reductase; SAD, stearoyl-ACP desaturase; FAD6, oleate desaturase (chloroplast); FAD7, linoleate desaturase (chloroplast); FAD2, oleate desaturase (endoplasmic reticulum); FAD3, linoleate desaturase (microsomal); FATA/B, acyl-ACP thioesterase A/B; PCH, palmitoyl-CoA hydrolase; LACS, long-chain Acyl-CoA synthetase; ACBP, acyl-CoA-binding protein; GPDH, NAD-dependent glycerol-3-phosphate dehydrogenase; CK, choline kinase; GPAT, glycerol-3-phosphate acyltransferase; LPAAT, 1-acylglycerol-3-phosphate acyltransferase; PAP, phosphatidic acid phosphatase; DGAT1/2, acyl-CoA: diacylglycerol acyltransferase 1/2; PLA2/C, phospholipase A2/C; LPCAT, 1-acylglycerol-3-phosphocholine acyltransferase; PDAT, phospholipid:diacylglycerol acyltransferase; CCT, cholinephosphate cytidylyltransferase; DAG-CPT, diacylglycerol cholinephosphotransferase; OBO, oil-body oleosin; STERO, steroleosin.

In total, 397 unigenes involved in FAs biosynthesis and TAG assembly were identified (see Additional file 4). In the FAs biosynthesis pathway, 97 genes involved in the initiation and acyl chain elongation steps of *de novo* FAs biosynthesis were identified, including 38 unigenes encoding pyruvate dehydrogenase complex (PDHC) subunits (seven for α-PDHC, six for β-PDHC, 11 for dihydrolipoamide acetyltransferase, and 14 for dihydrolipoamide dehydrogenase, respectively), 19 unigenes encoding acetyl-CoA carboxylase (ACCase) subunits (five for α-carboxyltransferase, six for β-carboxyltransferase, two for biotin carboxylase, and six biotin carboxyl carrier protein, respectively), one unigene encoding malonyl-CoA ACP transacylase (MCAAT), 13 unigenes encoding 3-ketoacyl-ACP synthases (KAS) (10 for KAS I, one for KAS II, and two for KAS III, respectively), and six unigenes encoding other components of FA synthase (one unigene encoding hydroxyacyl-ACP dehydrase HAD, two unigenes encoding ketoacyl-ACP reductase KAR, and three unigenes encoding enoyl-ACP reductase EAR) (see Figure 8A). The RPKM values showed that these genes involved in the initiation of *de novo* FA biosynthesis had at least one isoform significantly upregulated in the fast oil accumulation stage compared with the initial stage of seed development (see Additional file 4, Initiation), whereas all those genes involved in acyl chain elongation of FAs were upregulated in the fast oil accumulation stage, suggesting their functional involvement in FAs biosynthesis (see Additional file 4, Elongation). In addition, we identified eight unigenes encoding enzymes catalysing reactions that ultimately produce free FAs (two for acyl-ACP thioesterase A FATA, three for acyl-ACP thioesterase B FATB, and three for palmitoyl-CoA hydrolase PCH), 41 unigenes encoding long-chain acyl-CoA synthetases (LACS, which catalyzes esterification of free FAs to CoA upon arrival in the cytoplasm), and seven unigenes encoding acyl-CoA binding proteins (ACBP, which transports acyl-CoAs between organelles) (see Figure 8A). Both unigene 24779 and unigene 8250 encoding FATA were upregulated 4-fold, and unigenes 10273, 23453, 6530, 65564, 65528, 68727 and 66019 encoding LACS, were upregulated more than 30-fold in the fast oil accumulation stage (see Additional file 4, Thioesterase), suggesting their involvement in FA synthesis.

In the pathway of TAG assembly (Kennedy pathway), 77 unigenes were identified, including 14 unigenes encoding glycerol-3-phosphate acyltransferase (GPAT, which catalyzes the initial step of the Kennedy pathway), 14 unigenes encoding lysophosphatidic acid acyltransferase (LPAAT, which catalyzes the acylation of *sn*-1-acyl lysophosphatidic acid to form phosphatidic acid), 25 unigenes encoding phosphatidic acid phosphatase (PAP, which converts phosphatidic acid to sn-1, 2-diacylglycerol), 13 unigenes encoding acyl-CoA: diacylglycerol acyltransferase (DGAT, which transfer an acyl group from acyl-CoA to the sn-3 position of sn-1, 2-diacylglycerol to form TAG) (see Figure 8B). Besides, 11 unigenes encoding NAD-dependent glycerol-3-phosphate dehydrogenase (GPDH, which catalyzes sn-glycerol 3-phosphate, an initial substrate for Kennedy pathway) were identified (see Figure 8B). In the fast oil accumulation stage, expression of unigene21846 and unigene33663 encoding GPDH were upregulated more than 8-fold; expression of unigene280 encoding PAP was upregulated 7-fold; expression of unigene30542 encoding DGAT1 was upregulated 7-fold; expression of all unigenes encoding DGAT2 were substantially upregulated (see Additional file 4, Kennedy pathway). These results strongly implied that these genes upregulated in the fast oil accumulation stage might play a major role in TAG assembly. After biosynthesis, pools of TAG usually are stored in the form of oil bodies surrounded by oleosin or steroleosin proteins in seeds. Generally, the expression of oleosin genes is tightly associated with oil accumulation in developing seeds [52]. Seven unigenes encoding oil-body oleosins and seven unigenes encoding steroleosins (see Figure 8B) were identified with high expression in the fast oil accumulation stage (see Additional file 4, Oil accumulation), suggesting their involvement in oil accumulation in Sacha Inchi seeds.

For the formation of unsaturated FAs, 21 unigenes encoding fatty acid desaturase were identified, including three unigenes encoding stearoyl-ACP desaturase [SAD, which removes two hydrogen atoms from stearic acid (18C:0) to form oleic acid (18C:1)] (Figure 8A), eight unigenes encoding oleate desaturase [two for FAD6 and six for FAD2, both of which remove two hydrogen atoms from oleic acid to form linoleic acid (18C:2)] (Figure 8A,B), and ten unigenes encoding linoleate desaturase [(eight for FAD7 and two for FAD3, both of which remove two hydrogen atoms from linoleic acid to form linolenic acid (18C:3)] (Figure 8A,B). Because the oleic acid, linoleic acid and linolenic acid are the major constituents of PUFAs in Sacha Inchi seeds, the function of 21 unigenes identified may underlie the molecular basis of PUFAs formation in Sacha Inchi seeds. Expression analysis of the 21 unigenes between the initial and the fast oil accumulation stages showed that all unigenes encoding FAD6 and FAD7 genes (both of which were located and expressed in plastids in plant cells) were weakly expressed in the fast oil accumulation stage, while most of unigenes encoding SAD, FAD2 and FAD3 genes were highly expressed in the fast oil accumulation stage (see Additional file 4, Desaturation). In particular, the expression of unigene 15582 encoding FAD2 was upregulated 29-fold, and expression of unigene 17787 and unigene 54197 encoding FAD3 were upregulated 265-fold and 287-fold respectively (see Additional file 4, Desaturation). Genes FAD2 and FAD3 were both located and expressed in ER in cells in association with storage lipid accumulation in cells [15]. These results indicated that genes FAD2 and FAD3 might play a major role in the formation of PUFAs in Sacha Inchi seeds. Further cloning and functional analyses for unigenes 15582, 17787 and 54197 would probably reveal the molecular mechanisms underlying PUFAs biosynthesis in Sacha Inchi seeds. In addition, Acyl-CoA independent TAG assembly pathway including acyl editing and PC-DAG interconversion was believed to facilitate the incorporation of polyunsaturated FAs into TAG in some plant species [53]. 97 unigenes encoding phospholipases and 25 unigenes related to the Acyl-CoA independent TAG biosynthesis pathway were identified (see Figure 8B and Additional file 4). All of the twenty-five unigenes, however, have low transcript levels in the fast oil accumulation stage (see Additional file 4, Acyl editing/alternative TAG synthesis), and transcripts coding for phosphatidylcholine: diacylglycerol cholinephosphotransferase (PDCT), a key enzyme in PUFA synthesis in the Acyl-CoA independent TAG-synthetic pathway [29], was not detected in our libraries. These results implied that the Acyl-CoA independent TAG biosynthesis pathway might not be an active pathway in TAG biosynthesis in Sacha Inchi seeds. For the 97 unigenes encoding phospholipases, most of the unigenes have relatively higher transcript levels in the initial stage of seed development compared to the fast oil accumulation stage (see Additional file 4, Phospholipases). These unigenes may be related to the formation of membrane lipids and cell division. Lipases that arose in developing seeds of castor bean may be involved in re-modelling of TAGs after synthesis [54,55]. Seventeen unigenes encoding lipases (nine for monoglyceride lipase and eight for triacylglycerol lipase) were identified from our libraries. The function of these lipase genes in Sacha Inchi developing seeds is unclear. The unigenes 11440 and 1193 encoding TAG lipases were upregulated 13-fold and 7-fold in the fast oil accumulation stage, respectively (see Additional file 4, TAG lipases). It appears reasonable to assume these lipase unigenes were involved in re-modelling of TAGs after synthesis in Sacha Inchi seeds.

## Conclusions

We report here the first comprehensive dataset derived by using high-throughput sequencing technology (Illumina) for Sacha inchi. Transcriptome analyses from two developmental stages (the initial stage of seed development and the fast oil accumulation stage) of Sacha Inchi seeds revealed 70,392 unigenes. Expression of 22,179 unigenes differed at least 2-fold between the two libraries. Transcriptome sequences were annotated with Gene Ontology and KEGG Orthology identifiers. Functional categorization of differentially expressed genes reflected a number of important pathways, including the *de novo* FA biosynthesis, TAG assembly and the formation of PUFAs. Focusing on lipid genes, we identified 397 unigenes associated with the *de novo* FA biosynthesis, TAG assembly and the formation of PUFAs from Sacha Inchi seeds. These unigenes provide the foundation for further studies on molecular mechanisms underlying oil accumulation and PUFA biosynthesis in Sacha Inchi seeds. In particular, those candidate genes (such as SAD, FAD2, FAD3) associated with PUFA biosynthesis would provide critical clues to reveal the molecular mechanisms underlying the high levels of α-linolenic acid biosynthesis in Sacha Inchi seeds.

## Methods

### Biological material and RNA preparation

The two-year-old Sacha Inchi trees introduced from Peru by seeds were grown at Xishuangbanna Tropical Botanical Garden (21°56^′^ N, 101°15^′^ E, 600 m asl) at the Chinese Academy of Sciences, Yunnan, China under natural climate conditions. We observed the development process of Sacha Inchi fruits and seeds from female flowers pollinated to mature seeds in March-October, 2011. Mature female flowers were tagged and hand-pollinated by the time when the stigma was fully expanded, and the tagging dates were recorded as 0 day after pollination (DAP). Capsules at different development stages were harvested and dissected, and the oil content of developing seeds were measured as described previously by Xu et al. [56]. Based on the changes of oil accumulation in developing seeds (data not shown), we determined two stages for transcriptome sampling, i.e. the initial stage of seed development (S1, 5–10 days DAP) and in the fast oil accumulation stage (S2, 50–65 DAP). The developing seeds did not start to accumulate TAG in S1 stage and fast accumulated TAG in S2 stage (Figure 1). For the transcriptome sampling, the seeds at two developing stages were collected from the same individual at the same time (at ca 10:00 a.m.) of a day (on September 12, 2011). The collected samples were flash frozen in liquid nitrogen and stored at −80°C until further use. Total RNA from the two seed groups was prepared using Trizol reagent (Invitrogen, USA) and purified by using an RNeasy Mini Kit (Qiagen, Hilden, Germany) according to the manufacturers’ protocols. RNA integrity was evaluated with a 1.0% agarose gel stained with Goldview. Then RNA samples were quantified and examined for protein contamination (A_260_/A_280_) and reagent contamination (A_260_/A_230_) by using a NanoDrop ND-1000 spectrophotometer. The RNA integrity number determined by the Agilent 2100 Bioanalyzer was greater than 9.0 for both samples.

### cDNA library preparation and sequencing

Beads with oligo (dT) were used to isolate poly (A) mRNA from total RNA, according to the Illumina manufacturer’s instructions. Fragmentation buffer was added to disrupt the mRNA into short fragments. Using these short fragments as templates, random hexamers were used as primers to synthesize first-strand cDNA. Second-strand cDNA was synthesized using buffer, dNTPs, RNaseH, and DNA polymerase I. Short fragments were purified with QIAquick PCR extraction kit and resolved with EB buffer for end repair and poly (A) addition. Then, the short fragments were connected with sequencing adapters. After agarose gel electrophoresis, suitable fragments were selected as templates for PCR amplification. The cDNA library was sequenced at The Beijing Genome Institute (Shenzhen, China) using an Illumina HiSeq™ 2000 sequencing system.

### Analysis of Illumina sequencing results

Sequencing-received raw image data were transformed by base calling into sequence data. Raw reads with only 3' adaptor fragments were removed before data analysis. Clean reads were assembled using the short reads assembly program SOAPdenovo [44] and clustered using TGI Clustering tools [57]. Unigenes were aligned by BLASTX (e-value < 0.00001) to the NCBI nonredundant, SWISS-PROT, and KEGG protein databases. Unigenes that could not be aligned with any database were scanned by ESTScan [45]. We used the Blast2GO program [46] to determine Gene Ontology annotation of unigenes. After annotating every unigene, we used WEGO software [58] to determine the Gene Ontology-based functional classification for all unigenes. KEGG Orthology annotations of the unigenes were determined using InterProScan software.

### Differential expression of unigenes

To compare differences in gene expression, tag frequencies of both libraries were analyzed according to the method described by Audic and Claverie [59]. The false discovery rate was used to determine the threshold P-value for multiple testing. False discovery rate <0.001 and absolute value of the log_2_ ratio >1 were used as the threshold to determine significant differences in gene expression. Calculation of unigene expression uses the RPKM method [36]; the formula is shown below:

(1) $$\;\mathrm{RPKM}=\frac{10^{6}C}{\mathrm{NL}/10^{3}}$$

Set RPKM (A) as the expression of unigene A, C as the number of reads uniquely aligned to unigene A, N as the total number of reads uniquely aligned to all unigenes, and L as the number of bases in unigene A. The RPKM method eliminates the influence of gene length and sequencing level on the calculation of gene expression. The calculated gene expression can thus be directly used to compare gene expression between samples.

### Gene Ontology and KEGG Orthology enrichment analyses for differentially expressed unigenes

Gene Ontology and KEGG Orthology enrichment analyses of the differentially expressed genes were then carried out. Enriched P-values were calculated according to the hypergeometric test:

(2) $$\;P=1-\sum_{i=0}^{m-1}\frac{\left(\begin{array}{c} M\\ i \end{array}\right)\;\left(\begin{array}{c} N-M\\ n-i \end{array}\right)}{\left(\begin{array}{c} N\\ n \end{array}\right)}$$

In this equation, N represents the number of genes with Gene Ontology/KEGG Orthology annotation, n represents the number of differentially expressed genes in N, M represents the number of genes in each Gene Ontology/KEGG Orthology term, and m represents the number of differentially expressed gene in each term. For Gene Ontology enrichment analysis, all P-values were adjusted with the Bonferroni correction. We selected the corrected P-value 0.05 as the threshold to determine significant enrichment of the gene sets. For KEGG Orthology enrichment analysis, we used the false discovery rate 0.05 as the threshold to determine significant enrichment of the gene sets.

## Abbreviations

FA: Fatty acid; TAG: Triacylglycerol; ACP: Acyl carrier protein; DAG: Diacylglycerol; PC: Phosphatidylcholine; PUFA: Polyunsaturated fatty acid; DAP: Days after pollination; NCBI: National Center for Biotechnology Information; KEGG: Kyoto Encyclopedia of Genes and Genomes; RPKM: Reads per kb per million reads; PDHC: Pyruvate dehydrogenase; KAS: 3-ketoacyl-acyl carrier protein synthase; SAD: Stearoyl-ACP desaturase; GPAT: Glycerol-3-phosphate acyltransferase; LPAAT: Lysophosphatidic acid acyltransferase; DGAT: Diacylglycerol acyltransferase.

## Competing interests

The authors declare that we have no competing interests.

## Authors’ contributions

XW carried out sequence data analysis and drafted the manuscript. RX contributed to sample collection, RNA extraction, and manuscript revision. RW assisted with the oil extraction. AL designed and managed the experiments, and organized the manuscript. All authors read and approved the final version of the manuscript.

## Supplementary Material

Additional file 1 KEGG categories of nonredundant unigenes in Sacha Inchi. Click here for file

Additional file 2 Gene Ontology categories of unigenes with significant transcriptional changes during different stages of seed development. Click here for file

Additional file 3 KEGG Orthology enrichment analysis of unigenes with significant transcriptional changes during different stages of seed development. Click here for file

Additional file 4 Enzymes/proteins related to lipid accumulation in Sacha Inchi seeds. Click here for file
