# Supplementary material for: Transcriptome analysis of Sacha Inchi (Plukenetia volubilis L.) seeds at two developmental stages
Source: BMC Genomics. 2012 Dec 20;13:716. doi: 10.1186/1471-2164-13-716 (PMC3574040; doi:10.1186/1471-2164-13-716)
Supplement: Additional file 1 — KEGG categories of nonredundant unigenes in Sacha Inchi. [file 1471-2164-13-716-S1.doc]

**Additional file 1 :**

**KEGG categories of nonredundant Unigenes in Sacha inchi**

| **#** | **Pathway** | **Count (20142)** | **Pathway ID** |
| --- | --- | --- | --- |
| 1 | Metabolic pathways | 4360 | ko01100 |
| 2 | Biosynthesis of secondary metabolites | 2348 | ko01110 |
| 3 | Plant-pathogen interaction | 1711 | ko04626 |
| 4 | Spliceosome | 994 | ko03040 |
| 5 | Ribosome | 684 | ko03010 |
| 6 | Starch and sucrose metabolism | 579 | ko00500 |
| 7 | Protein processing in endoplasmic reticulum | 525 | ko04141 |
| 8 | Phenylpropanoid biosynthesis | 482 | ko00940 |
| 9 | Purine metabolism | 437 | ko00230 |
| 10 | Ubiquitin mediated proteolysis | 417 | ko04120 |
| 11 | Oxidative phosphorylation | 373 | ko00190 |
| 12 | Pyrimidine metabolism | 349 | ko00240 |
| 13 | Glycolysis / Gluconeogenesis | 322 | ko00010 |
| 14 | Cysteine and methionine metabolism | 300 | ko00270 |
| 15 | Endocytosis | 294 | ko04144 |
| 16 | Peroxisome | 291 | ko04146 |
| 17 | RNA degradation | 275 | ko03018 |
| 18 | Stilbenoid, diarylheptanoid and gingerol biosynthesis | 265 | ko00945 |
| 19 | Limonene and pinene degradation | 262 | ko00903 |
| 20 | Circadian rhythm - plant | 244 | ko04712 |
| 21 | Phagosome | 235 | ko04145 |
| 22 | Flavonoid biosynthesis | 233 | ko00941 |
| 23 | Nucleotide excision repair | 228 | ko03420 |
| 24 | Pyruvate metabolism | 222 | ko00620 |
| 25 | Amino sugar and nucleotide sugar metabolism | 218 | ko00520 |
| 26 | Carbon fixation in photosynthetic organisms | 210 | ko00710 |
| 27 | Cyanoamino acid metabolism | 206 | ko00460 |
| 28 | Phenylalanine metabolism | 192 | ko00360 |
| 29 | ABC transporters | 189 | ko02010 |
| 30 | alpha-Linolenic acid metabolism | 180 | ko00592 |
| 31 | Glycerophospholipid metabolism | 179 | ko00564 |
| 32 | Pentose and glucuronate interconversions | 177 | ko00040 |
| 33 | Glutathione metabolism | 168 | ko00480 |
| 34 | Arginine and proline metabolism | 167 | ko00330 |
| 35 | Fatty acid metabolism | 163 | ko00071 |
| 36 | Tryptophan metabolism | 163 | ko00380 |
| 37 | Zeatin biosynthesis | 159 | ko00908 |
| 38 | Aminoacyl-tRNA biosynthesis | 158 | ko00970 |
| 39 | DNA replication | 158 | ko03030 |
| 40 | Basal transcription factors | 156 | ko03022 |
| 41 | Proteasome | 156 | ko03050 |
| 42 | Fructose and mannose metabolism | 148 | ko00051 |
| 43 | Glycerolipid metabolism | 146 | ko00561 |
| 44 | Galactose metabolism | 145 | ko00052 |
| 45 | Phosphatidylinositol signaling system | 142 | ko04070 |
| 46 | Alanine, aspartate and glutamate metabolism | 141 | ko00250 |
| 47 | Nitrogen metabolism | 137 | ko00910 |
| 48 | RNA polymerase | 137 | ko03020 |
| 49 | Glycine, serine and threonine metabolism | 130 | ko00260 |
| 50 | Citrate cycle (TCA cycle) | 128 | ko00020 |
| 51 | Base excision repair | 127 | ko03410 |
| 52 | Pentose phosphate pathway | 126 | ko00030 |
| 53 | Homologous recombination | 125 | ko03440 |
| 54 | Porphyrin and chlorophyll metabolism | 124 | ko00860 |
| 55 | Inositol phosphate metabolism | 124 | ko00562 |
| 56 | Carotenoid biosynthesis | 121 | ko00906 |
| 57 | Biosynthesis of unsaturated fatty acids | 117 | ko01040 |
| 58 | Valine, leucine and isoleucine degradation | 116 | ko00280 |
| 59 | Linoleic acid metabolism | 114 | ko00591 |
| 60 | Butanoate metabolism | 114 | ko00650 |
| 61 | Protein export | 113 | ko03060 |
| 62 | Phenylalanine, tyrosine and tryptophan biosynthesis | 112 | ko00400 |
| 63 | Tyrosine metabolism | 112 | ko00350 |
| 64 | Natural killer cell mediated cytotoxicity | 111 | ko04650 |
| 65 | Photosynthesis | 111 | ko00195 |
| 66 | Terpenoid backbone biosynthesis | 111 | ko00900 |
| 67 | SNARE interactions in vesicular transport | 110 | ko04130 |
| 68 | Selenoamino acid metabolism | 107 | ko00450 |
| 69 | N-Glycan biosynthesis | 107 | ko00510 |
| 70 | Mismatch repair | 106 | ko03430 |
| 71 | Ascorbate and aldarate metabolism | 104 | ko00053 |
| 72 | Propanoate metabolism | 100 | ko00640 |
| 73 | Valine, leucine and isoleucine biosynthesis | 96 | ko00290 |
| 74 | Lysine degradation | 94 | ko00310 |
| 75 | Ubiquinone and other terpenoid-quinone biosynthesis | 94 | ko00130 |
| 76 | Regulation of autophagy | 90 | ko04140 |
| 77 | Fatty acid biosynthesis | 89 | ko00061 |
| 78 | beta-Alanine metabolism | 87 | ko00410 |
| 79 | Steroid biosynthesis | 86 | ko00100 |
| 80 | Glyoxylate and dicarboxylate metabolism | 84 | ko00630 |
| 81 | Sphingolipid metabolism | 80 | ko00600 |
| 82 | Ether lipid metabolism | 79 | ko00565 |
| 83 | Diterpenoid biosynthesis | 79 | ko00904 |
| 84 | Flavone and flavonol biosynthesis | 76 | ko00944 |
| 85 | Other glycan degradation | 75 | ko00511 |
| 86 | Sulfur metabolism | 69 | ko00920 |
| 87 | Pantothenate and CoA biosynthesis | 68 | ko00770 |
| 88 | Glucosinolate biosynthesis | 67 | ko00966 |
| 89 | Nicotinate and nicotinamide metabolism | 63 | ko00760 |
| 90 | Histidine metabolism | 55 | ko00340 |
| 91 | One carbon pool by folate | 54 | ko00670 |
| 92 | Glycosaminoglycan degradation | 42 | ko00531 |
| 93 | Folate biosynthesis | 41 | ko00790 |
| 94 | Benzoxazinoid biosynthesis | 39 | ko00402 |
| 95 | Tropane, piperidine and pyridine alkaloid biosynthesis | 37 | ko00960 |
| 96 | Non-homologous end-joining | 35 | ko03450 |
| 97 | Anthocyanin biosynthesis | 34 | ko00942 |
| 98 | Arachidonic acid metabolism | 33 | ko00590 |
| 99 | Lysine biosynthesis | 33 | ko00300 |
| 100 | Isoquinoline alkaloid biosynthesis | 32 | ko00950 |
| 101 | Photosynthesis - antenna proteins | 30 | ko00196 |
| 102 | Monoterpenoid biosynthesis | 30 | ko00902 |
| 103 | Thiamine metabolism | 30 | ko00730 |
| 104 | Riboflavin metabolism | 28 | ko00740 |
| 105 | Glycosylphosphatidylinositol(GPI)-anchor biosynthesis | 27 | ko00563 |
| 106 | Taurine and hypotaurine metabolism | 24 | ko00430 |
| 107 | Glycosphingolipid biosynthesis - ganglio series | 23 | ko00604 |
| 108 | Indole alkaloid biosynthesis | 23 | ko00901 |
| 109 | Brassinosteroid biosynthesis | 22 | ko00905 |
| 110 | Polyketide sugar unit biosynthesis | 22 | ko00523 |
| 111 | Vitamin B6 metabolism | 20 | ko00750 |
| 112 | Caffeine metabolism | 19 | ko00232 |
| 113 | Glycosphingolipid biosynthesis - globo series | 17 | ko00603 |
| 114 | Lipoic acid metabolism | 15 | ko00785 |
| 115 | Synthesis and degradation of ketone bodies | 15 | ko00072 |
| 116 | Biotin metabolism | 15 | ko00780 |
| 117 | Fatty acid elongation in mitochondria | 14 | ko00062 |
| 118 | C5-Branched dibasic acid metabolism | 12 | ko00660 |
| 119 | Betalain biosynthesis | 5 | ko00965 |
