# Supplementary figures and images for: Transcriptome analysis of Sacha Inchi (Plukenetia volubilis L.) seeds at two developmental stages
Source: BMC Genomics. 2012 Dec 20;13:716. doi: 10.1186/1471-2164-13-716 (PMC3574040; doi:10.1186/1471-2164-13-716)

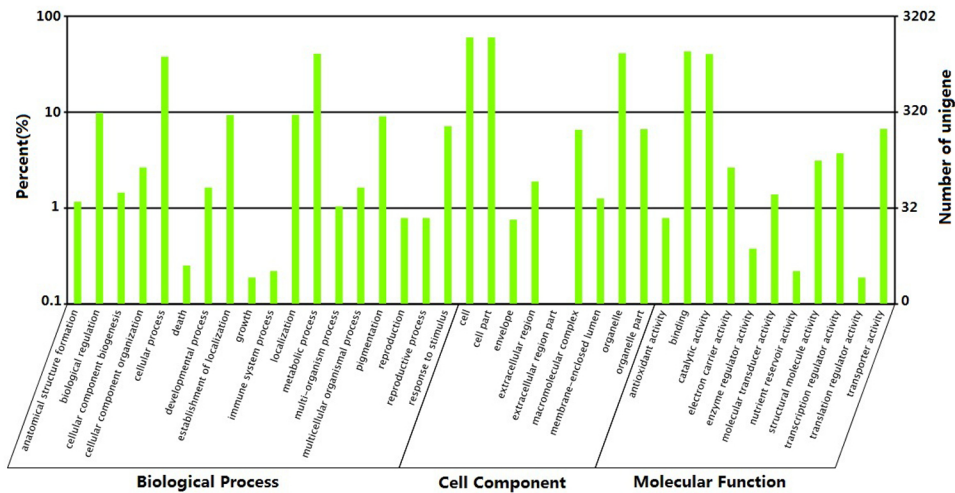

Supplement: Additional file 2 — Gene Ontology categories of unigenes with significant transcriptional changes during different stages of seed development. [file 1471-2164-13-716-S2.pdf]
