# Supplementary material for: Transcriptome analysis of Sacha Inchi (Plukenetia volubilis L.) seeds at two developmental stages
Source: BMC Genomics. 2012 Dec 20;13:716. doi: 10.1186/1471-2164-13-716 (PMC3574040; doi:10.1186/1471-2164-13-716)
Supplement: Additional file 3 — KEGG Orthology enrichment analysis of unigenes with significant transcriptional changes during different stages of seed development. [file 1471-2164-13-716-S3.doc]

**Additional file 3 :**

**KO enrichment analysis of Unigenes with significant transcriptional changes between between Sacha inchi seeds at different developmental stages.**

| **#** | **KEGG Pathway Category** | **DEGs^a^** | **FDR** | **Pathway ID** |
| --- | --- | --- | --- | --- |
| 1 | Plant-pathogen interaction | 1028 | 1.92E-75 | ko04626 |
| 2 | Biosynthesis of secondary metabolites | 1184 | 1.30E-32 | ko01110 |
| 3 | Phenylpropanoid biosynthesis | 306 | 4.80E-27 | ko00940 |
| 4 | Metabolic pathways | 1999 | 8.74E-26 | ko01100 |
| 5 | Starch and sucrose metabolism | 334 | 1.92E-19 | ko00500 |
| 6 | Phenylalanine metabolism | 125 | 2.28E-12 | ko00360 |
| 7 | Flavonoid biosynthesis | 145 | 4.96E-12 | ko00941 |
| 8 | Stilbenoid, diarylheptanoid and gingerol biosynthesis | 158 | 6.06E-11 | ko00945 |
| 9 | Monoterpenoid biosynthesis | 29 | 2.80E-10 | ko00902 |
| 10 | alpha-Linolenic acid metabolism | 112 | 1.72E-09 | ko00592 |
| 11 | Cysteine and methionine metabolism | 168 | 9.60E-09 | ko00270 |
| 12 | Nitrogen metabolism | 87 | 3.75E-08 | ko00910 |
| 13 | Linoleic acid metabolism | 75 | 4.12E-08 | ko00591 |
| 14 | Photosynthesis - antenna proteins | 27 | 6.42E-08 | ko00196 |
| 15 | Biosynthesis of unsaturated fatty acids | 75 | 1.94E-07 | ko01040 |
| 16 | Fatty acid biosynthesis | 60 | 2.99E-07 | ko00061 |
| 17 | Cyanoamino acid metabolism | 118 | 3.55E-07 | ko00460 |
| 18 | Diterpenoid biosynthesis | 54 | 6.16E-07 | ko00904 |
| 19 | Flavone and flavonol biosynthesis | 52 | 9.59E-07 | ko00944 |
| 20 | Limonene and pinene degradation | 139 | 1.00E-05 | ko00903 |
| 21 | Anthocyanin biosynthesis | 25 | 2.24E-04 | ko00942 |
| 22 | Terpenoid backbone biosynthesis | 64 | 2.24E-04 | ko00900 |
| 23 | Pentose and glucuronate interconversions | 94 | 3.71E-04 | ko00040 |
| 24 | Circadian rhythm - plant | 124 | 3.86E-04 | ko04712 |
| 25 | Ubiquinone and other terpenoid-quinone biosynthesis | 55 | 3.86E-04 | ko00130 |
| 26 | Galactose metabolism | 78 | 7.55E-04 | ko00052 |
| 27 | Zeatin biosynthesis | 84 | 9.36E-04 | ko00908 |
| 28 | Ascorbate and aldarate metabolism | 58 | 1.35E-03 | ko00053 |
| 29 | Tryptophan metabolism | 85 | 1.41E-03 | ko00380 |
| 30 | Alanine, aspartate and glutamate metabolism | 74 | 2.50E-03 | ko00250 |
| 31 | Fatty acid metabolism | 83 | 3.98E-03 | ko00071 |
| 32 | Taurine and hypotaurine metabolism | 17 | 5.44E-03 | ko00430 |
| 33 | Ether lipid metabolism | 44 | 5.98E-03 | ko00565 |
| 34 | Glyoxylate and dicarboxylate metabolism | 46 | 7.34E-03 | ko00630 |
| 35 | Carbon fixation in photosynthetic organisms | 102 | 7.89E-03 | ko00710 |
| 36 | Glycolysis / Gluconeogenesis | 150 | 7.95E-03 | ko00010 |
| 37 | Glucosinolate biosynthesis | 37 | 1.46E-02 | ko00966 |
| 38 | Brassinosteroid biosynthesis | 15 | 1.56E-02 | ko00905 |
| 39 | Glycerolipid metabolism | 72 | 1.82E-02 | ko00561 |
| 40 | ABC transporters | 90 | 2.36E-02 | ko02010 |
| 41 | Amino sugar and nucleotide sugar metabolism | 101 | 3.86E-02 | ko00520 |
| 42 | Isoquinoline alkaloid biosynthesis | 19 | 4.05E-02 | ko00950 |
| 43 | Arginine and proline metabolism | 79 | 4.05E-02 | ko00330 |
| 44 | Selenoamino acid metabolism | 53 | 4.05E-02 | ko00450 |
| 45 | Pyruvate metabolism | 102 | 4.47E-02 | ko00620 |

^a^DEGs= Differentially Expressed Unigenes
